# How can we better support the public health emergency response workforce during crises?

**DOI:** 10.5365/wpsar.2021.12.4.886

**Published:** 2021-11-23

**Authors:** Amy Elizabeth Parry, Samantha M Colquhoun, Emma Field, Martyn D Kirk, David N Durrheim, Tambri Housen

**Affiliations:** a Australian National University, Australian Capital Territory, Australia.; b University of Newcastle, New South Wales, Australia.

The public health emergency response workforce has experienced unrelenting pressure during the past decade. Countries in the Western Pacific Region have responded to significant outbreaks of avian influenza, Zika virus disease, Middle East respiratory syndrome, vaccine-derived poliovirus, measles and the coronavirus disease 2019 (COVID-19) pandemic, as well as natural disasters; they also supported the response to Ebola virus disease in West Africa during 2014–2016. (*1*) For public health responses to be effective, we must continue to identify optimal mechanisms to support people working in challenging public health responses.

Health systems strengthening, in particular for workforce support, is fundamental to achieving the core capacity required under the International Health Regulations (2005). (*2*) The Asia Pacific Strategy for Emerging Diseases and Public Health Emergencies (APSED III) recognizes that a skilled, experienced local public health workforce must be developed and maintained to prevent the escalation and spread of emergencies. (*3*)

The IHR Joint External Evaluations show that work remains to be done to strengthen public health workforces so that they can manage health security events. (*4*) The COVID-19 pandemic has clearly demonstrated that large public health events require responders with skills and expertise to address the crisis appropriately. In May 2021, the World Health Assembly recommended investment in the health workforce for better management of the COVID-19 pandemic. (*5*)

In the Western Pacific Region, field epidemiology training programmes (FETPs) are a key activity for strengthening health security by developing vital technical expertise in the existing workforce. (*3*, *6*) The programmes are based on the principle of “learning through doing” with guidance from experienced epidemiologists. (*6*) Such support, however, often stops at graduation. A guiding principle of APSED III is “continuous learning and improvement.” (*3*) Thus, preparedness before a crisis is an integral component, but professional support to the health workforce during crises would be feasible for consolidating what has been learnt.

In 2019, we interviewed public health emergency response experts on topics that included workforce support. The experts discussed the challenge of inexperience and noted that an emergency response surge workforce was frequently based on availability rather than appropriate skills and experience. (*7*) Less experienced epidemiologists were often readily available for rapid deployment, but emergency response was considered not to be an ideal training setting. The experts stated that less experienced responders could be considered suitable if they were guided. (*7*)

To support the technical and leadership needs of the surge workforce during the COVID-19 pandemic in Australia, the Public Health Association of Australia and the Australasian Epidemiological Association rapidly established a pilot mentorship programme for surge responders, in which mentors provided both professional and personal support to mentees remotely. (*8*, *9*) Subsequent evaluation showed that the programme effectively supported a workforce with limited prior public health experience to work in a stressful environment during a national crisis. The mentors were found to improve the confidence of the mentees in conducting their work by sharing their professional skills in areas such as leadership and decision-making. Importantly, the mentors supported the well-being of the mentees by acting as a confidential sounding board and guiding them in navigating political and otherwise complex environments. (*8*, *9*)

The Australian mentorship programme supported front-line pandemic surge response workers at a time of great need. The main recommendation of the evaluation was to design a purpose-built programme for supporting emergency response workers. (*8*, *9*) Difficulties associated with such support include the fact that people are involved in a response for only short periods and are often new to the context or organization in which they are working. Provision of support during emergencies can also be limited by lack of time and cross-cultural challenges.

**Figure 1:**
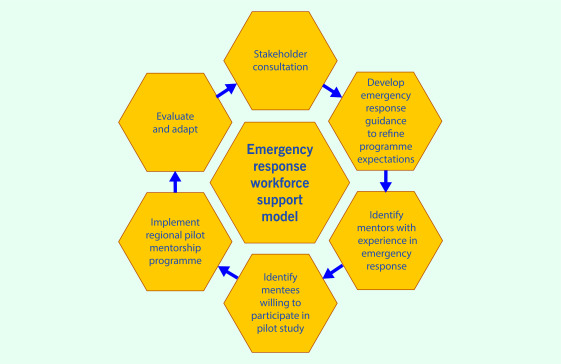
Recommended steps for establishing an emergency response workforce support model

A similar programme in the Western Pacific Region, based on the experience of the Australian programme, (*8*, *9*) could provide support for the COVID-19 response and also an opportunity to learn and prepare for future public health emergencies. Stakeholders such as partners in the Global Outbreak Alert and Response Network should be consulted to design an all-purpose emergency response support model and materials and to pilot-test the programme and evaluate comprehensively what works and how. The recommended steps in establishing a pilot programme are illustrated in **Fig. 1**.

Such a support programme could be used in public health emergency response both locally and globally. It could increase the effectiveness of the workforce, add to professional knowledge, provide less experienced responders with skills and reduce stress and burn-out. (*8*) The proposed pilot programme would also benefit long-term national and regional preparedness, providing individuals and countries with peer-supported learning and experience.

[**insert** Figure 1]

The first objective of the WHO Global Strategy on Human Resources for Health is to optimize the quality of performance and the impact of the workforce. (*10*) This should be based on emerging evidence on strengthening and continuing to support the health workforce during crises. To ensure that the Region becomes “the healthiest and safest,” (*11*) high-quality, longer-term programmes will be necessary, such as FETPs to ensure sustained workforce development. In crises, however, a mentoring-like programme might foster consistent support for and empowerment of the workforce.
